# Advancing Clostridia to Clinical Trial: Past Lessons and Recent Progress

**DOI:** 10.3390/cancers8070063

**Published:** 2016-06-28

**Authors:** Alexandra M. Mowday, Christopher P. Guise, David F. Ackerley, Nigel P. Minton, Philippe Lambin, Ludwig J. Dubois, Jan Theys, Jeff B. Smaill, Adam V. Patterson

**Affiliations:** 1 Translational Therapeutics Team, Auckland Cancer Society Research Centre, School of Medical Sciences, University of Auckland, Auckland 1023, New Zealand; a.mowday@auckland.ac.nz (A.M.M.); c.guise@auckland.ac.nz (C.P.G.); j.smaill@auckland.ac.nz (J.B.S.); 2 Maurice Wilkins Centre for Molecular Biodiscovery, School of Biological Sciences, University of Auckland, Auckland 1023, New Zealand; 3 School of Biological Sciences, Victoria University of Wellington, Wellington 6140, New Zealand; david.ackerley@vuw.ac.nz; 4 The Clostridia Research Group, BBSRC/EPSRC Synthetic Biology Research Centre (SBRC) School of Life Sciences, University of Nottingham, Nottingham NG72RD, UK; nigel.minton@nottingham.ac.uk; 5 Maastro (Maastricht Radiation Oncology), GROW School for Oncology and Development Biology, Maastricht University Medical Centre, 6200 MD Maastricht, The Netherlands; philippe.lambin@maastro.nl (P.L.); ludwig.dubois@maastrichtuniversity.nl (L.J.D.); jan.theys@maastrichtuniversity.nl (J.T.)

**Keywords:** *Clostridium*, cancer, gene therapy, imaging, prodrug, radiotherapy, immunotherapy

## Abstract

Most solid cancers contain regions of necrotic tissue. The extent of necrosis is associated with poor survival, most likely because it reflects aggressive tumour outgrowth and inflammation. Intravenously injected spores of anaerobic bacteria from the genus *Clostridium* infiltrate and selectively germinate in these necrotic regions, providing cancer-specific colonisation. The specificity of this system was first demonstrated over 60 years ago and evidence of colonisation has been confirmed in multiple tumour models. The use of “armed” clostridia, such as in *Clostridium* Directed Enzyme Prodrug Therapy (CDEPT), may help to overcome some of the described deficiencies of using wild-type clostridia for treatment of cancer, such as tumour regrowth from a well-vascularised outer rim of viable cells. Successful preclinical evaluation of a transferable gene that metabolises both clinical stage positron emission tomography (PET) imaging agents (for whole body vector visualisation) as well as chemotherapy prodrugs (for conditional enhancement of efficacy) would be a valuable early step towards the prospect of “armed” clostridia entering clinical evaluation. The ability to target the immunosuppressive hypoxic tumour microenvironment using CDEPT may offer potential for synergy with recently developed immunotherapy strategies. Ultimately, clostridia may be most efficacious when combined with conventional therapies, such as radiotherapy, that sterilise viable aerobic tumour cells.

## 1. Tumor Necrosis as a Target for Cancer Therapy

Solid tumours account for approximately 90% of all diagnosed cancer [1], and the microenvironment of these tumours generally contain a complex, disarrayed set of blood vessels due to aberrant tumour angiogenesis. These blood vessels are highly irregular, with arteriovenous shunts and blunt ends, incomplete endothelial linings resulting in increased vascular permeability, and irregular, sluggish blood flow [2]. As such, delivery of oxygen and nutrients to tumour tissue becomes much less efficient than usual, leading to low overall oxygen levels and areas of hypoxia. Tumour regions located further from a blood vessel than the diffusion limit of oxygen can become chronically hypoxic/anoxic and eventually necrotic. These necrotic regions are a typical, if not universal, histological feature of human solid tumours, and are largely associated with high-risk tumour characteristics [3]. There is now a substantial body of evidence confirming the poor prognostic value of tumour necrosis in human solid tumours, including in brain [4], breast [5], colorectal [6], melanoma [7], non-small cell lung [8], pancreatic [9], and renal cell [10] malignancies. Currently there are no clinically approved therapeutic interventions to exploit this phenomenon, yet necrosis offers the most desirable of all attributes for targeted therapy—absolute specificity for neoplasia—as it is categorically absent from healthy normal tissues.

Tumour necrosis is known to permit the growth of anaerobic bacteria such as clostridia, offering a unique opportunity to turn a pathological feature usually associated with treatment failure into a precision therapy. *Clostridium* is one of the largest prokaryotic genera, comprising a heterogeneous group of rod-shaped, anaerobic, spore-forming bacteria. In times of stress, they can undergo a complex cell differentiation process resulting in the production of endospores, rendering them highly resistant to harsh environmental conditions such as high temperature and dehydration [11]. Being obligate anaerobes, *Clostridium* spores germinate into metabolically active vegetative cells in the absence of oxygen. Furthermore, their saprophytic nature ensures they thrive in habitats that contain abundant organic matter [12]. The well-known pathogenic species *Clostridium tetani*, *Clostridium botulinum, Clostridium novyi,* and *Clostridium perfringens* germinate in necrotic tissue to produce toxins that cause respectively tetanus, botulism, haemolysis, and gas gangrene [13]. Except for these species, most members are non-pathogenic inhabitants of the soil.

## 2. Discovery and Early Development of *Clostridium* as an Anticancer Agent

Clostridia were first associated with cancer in 1813, when Vautier observed tumour regressions in patients who contracted gas gangrene after infection with *C. perfringens*. Over a century later, tumour lysis of a Flexner-Jobling rat carcinoma was reported after selectively growing different *Clostridium* species in the tumour [14]. Connell subsequently determined that the tumour regression observed after *Clostridium histolyticum* infection was due to the production of proteolytic enzymes preferentially degrading the tumour tissue without affecting normal tissue [15]. The oncolytic effects of *C. histolyticum* were tested further by injecting a spore suspension into transplanted sarcomas of mice. This resulted in tumour regression (liquefaction) and extended survival of the tumour bearing animals [16]. Few animals survived this treatment, however, as the oncolysis observed was accompanied by toxicity and death. In 1955 the specificity of the system was demonstrated using intravenously injected *C. tetani* spores [17]. Delivery of these spores to tumour bearing mice resulted in death from tetanus poisoning within 48 hours, whereas mice without tumours were able to clear the spores without side effects. Examination of tissues showed that the vegetative clostridial cells were localised to the tumour and could not be detected elsewhere in the body, confirming the specificity of germination and demonstrating that systemic administration of spores was sufficient for effective tumour colonisation.

It was then reasoned that a non-pathogenic soil isolate of clostridia, *Clostridium butyricum* M55, later renamed *Clostridium oncolyticum* and now classified as *Clostridium sporogenes* (ATCC13732), might have the same ability to cause tumour regression without causing toxicity-related death [18]. This *Clostridium* species was shown to localise and germinate in solid Erhlich tumours, causing extensive lysis (tumours first softened and later liquefied) with no effect on normal tissues. Not all mice survived this stage of extensive oncolysis, but those that did demonstrated tumour regrowth from the remaining outer rim of viable cells. Similar observations were made and extended for other non-pathogenic *Clostridium* species by a number of investigators [19,20,21]. Overall, these early studies indicated that germination of non-pathogenic clostridia was well tolerated in animal models and frequently resulted in the destruction of a significant portion of the tumour.

Mosë and Mosë then took the unprecedented step of demonstrating the absence of pathogenicity in the *C. butyricum* M55 strain by injecting themselves without harmful effects [22]. Following this, the first clinical trial was initiated in five patients with neoplastic disease [22]. After intravenous injection with 10^10^ *C. butyricum* M55 spores, oncolysis was observed in three patients with the largest tumours, but not in the surrounding tissues or smaller metastases. In one case, a transient clinical benefit was attributed to *Clostridium*-induced oncolysis. In 1978, Heppner and Mosë administered the same spore by intracarotid injection to patients with vascular glioblastomas [23]. One week after injection, complete oncolysis was observed in the majority of patients, with conversion of the glioblastoma into a brain abscess which was then operated on to prevent rupture and death. Patients had low-grade fever and blood cultures for approximately one week concomitant with tumour lysis, and occasionally required supportive care involving treatment with antibiotics. As a result of these studies it was apparent that although safety and evidence of colonisation following intravenous administration had been established, there was an overall lack of clinical benefit likely due to tumour regrowth from a well-vascularised outer rim of viable cells.

## 3. Recent Progress on the Use of Unarmed Clostridia to Treat Cancer

*C. novyi-NT* is a non-toxic derivative of the wild type *C. novyi* strain (with the lethal haemolysis causing α-toxin gene removed) and is one of the most clinically advanced *Clostridium* species to date. Pre-clinically, it has been shown that intravenously injected spores from *C. novyi-NT* are able to germinate in the necrotic regions of tumours in mice and destroy surrounding tumour cells [24]. In regard to anti-tumour efficacy, a 34% complete response (CR) was observed after intravenous injection of spores in the syngeneic mouse CT26 tumour model, with comparable efficacy findings (30% CR) in the rabbit hepatic VX2 tumour model [25]. In contrast, when companion dogs with spontaneously occurring tumours were treated by intravenous spore administration only stable disease was achieved at doses with acceptable toxicity [26]. One of the main dose limiting toxicities observed in the canine study was the development of tumour abscesses which required surgical intervention. Therefore, in a subsequent study also using dogs with spontaneously occurring tumours, intratumoural injection of spores was used instead of intravenous administration [27]. Three of the 14 dogs had a complete response (21% CR) to therapy and 3/14 had a partial response (PR). Of the six animals (43%) with objective responses, three had a long-term disease control. In this study the first human patient was also treated with *C. novyi-NT* after intratumoural injection and demonstrated colonisation of the tumour by *C. novyi-NT*, along with an absence of viable tumour cells by tissue biopsy. From these studies it appears that whilst *C. novyi-NT* demonstrated excellent tumour colonising properties, systemic administration is not always possible due to associated toxicity, limiting the ability to target tumours beyond the reach of percutaneous injection.

## 4. *Clostridium* Directed Enzyme Prodrug Therapy (CDEPT)

### 4.1. CDEPT Principle and Proof-of-Concept

**Figure 1:**
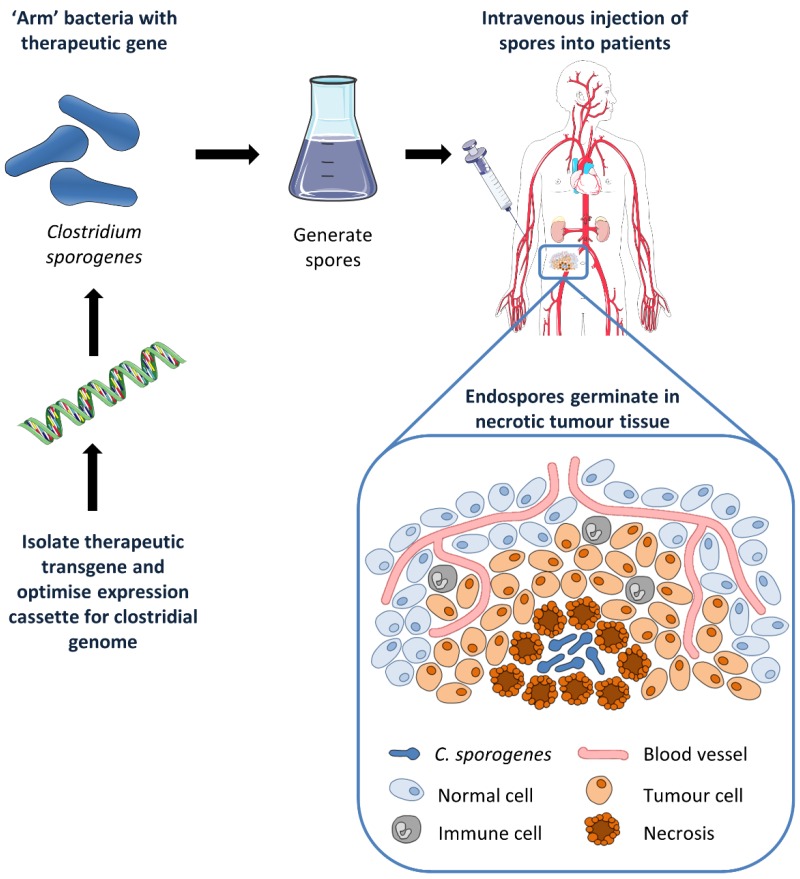
Cancer patients receive “armed” *C. sporogenes* spores which are able to germinate and colonise necrotic regions of their tumour.

Gene Directed Enzyme Prodrug Therapy (GDEPT) is a cancer gene therapy approach whereby an exogenous therapeutic gene introduces a new catalytic function specifically to a tumour cell, sensitising that cell to an otherwise inert prodrug [28]. The proposed *Clostridium* Directed Enzyme Prodrug Therapy (CDEPT) strategy is to inject spores of non-pathogenic clostridia that have been genetically modified to express a non-mammalian prodrug activating enzyme (Figure 1). Because germination to the vegetative form should only occur in the necrotic regions of the tumour, enzyme expression, and therefore prodrug metabolism, should also be restricted to the tumour, generating high concentrations of active drug specifically at the tumour site. If the activated drug has a large bystander effect (this being the ability of the activated drug to diffuse to and kill neighbouring cells), cell kill will be possible beyond the necrotic boundaries. As such, this approach was thought to have broad applicability to patients with solid tumours, and use of these genetically modified strains of clostridia may help to overcome some of the previously described drawbacks of using wild-type clostridia for the treatment of cancer.

**Figure 2:**
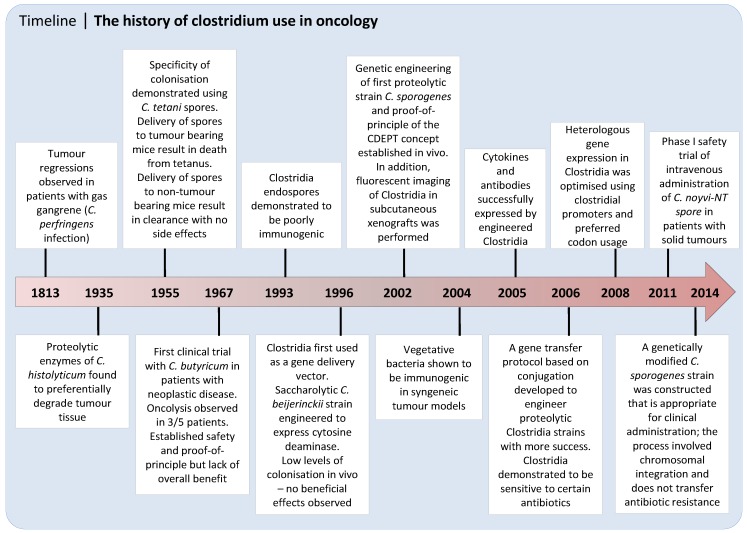
Key (pre)clinical steps in clostridial use and development. CDEPT, *Clostridium* Directed Enzyme Prodrug Therapy.

Different clostridial strains have highly diverse characteristics in terms of tumour colonisation and oncolytic capability, as well as predisposition to genetic engineering [12]. Initially, protocols for gene transformation were only available for saccharolytic strains and thus *Clostridium acetobutylicum* and *Clostridium beijerinckii* were among the first to be tested as gene delivery vectors (Figure 2). The principal effort was focused on the delivery of bacterial enzymes cytosine deaminase (CD) and the *E. coli* nitroreductase NfsB, both of which have essentially no human equivalent. CD activates the prodrug 5-fluorocytosine (5-FC) to generate the clinically used anti-metabolite 5-fluorouracil (5-FU), whereas *E. coli* NfsB can convert nitroaromatic prodrugs such as CB1954 into cytotoxic DNA damaging metabolites [29,30]. Fox and colleagues were first to clone CD into *C. beijerinckii* by use of a clostridial expression vector [31]. High levels of the active enzyme were found in the bacterial medium, and when this was added to an in vitro clonogenic assay, murine EMT6 tumour cells were sensitised to the prodrug 5-fluorocytosine up to 500-fold. Later, CD was expressed in *C. acetobutylicum* and tumour specific expression of CD was detected in subcutaneous rat rhabdomyosarcomas after intratumoural injection of spores [32]. Similar experiments were performed with NfsB expressed in *C. beijerinckii* [33]. In this study, intravenously injected spores produced detectable NfsB expression in 9/10 subcutaneous mouse EMT6 tumours during the first five days after administration.

Although recombinant enzyme activity was detected in these *Clostridium* colonised tumours, no studies reported any beneficial effects on tumour growth in vivo after administration of prodrug. The most likely explanation for this is that there were insufficient levels of enzyme in the tumour due to low levels of colonisation by these strains. Upon systemic administration of spores, tumour colonisation by saccharolytic clostridial strains has been shown to be up to two orders of magnitude lower than in comparable tumours colonised by proteolytic strains such as *C. sporogenes* [34]. The use of proteolytic strains of *Clostridium* for CDEPT applications had at this stage remained under-utilised due to their resistance to genetic engineering. A protocol for transformation of these strains was eventually developed using a polyethylene glycol-based transfection buffer containing DNAase inhibitors [34], but efficiency was extremely low and the method was not very reproducible. Nevertheless, Liu and colleagues managed to demonstrate CDEPT proof of principle using proteolytic clostridial strains. Mice bearing subcutaneous SCCVII tumours were intravenously injected with *C. sporogenes* (NCIMB10696) engineered to express CD. Active enzyme was produced only in the tumour, and systemic delivery of 5-fluorocytosine one day post spore injection produced a greater anti-tumour effect than maximally tolerated doses of 5-fluorouracil alone.

A major breakthrough was subsequently achieved using a gene transfer protocol based on conjugation [35], making it possible to genetically modify proteolytic clostridial strains that had improved tumour colonisation capacity (and therefore the highest gene expression levels) with high success rates. The NfsB homologue from *Haemophilus influenza*, *nfnB*, was expressed in *C. sporogenes* and colonisation of HCT116 tumours was achieved after spores were administered systemically to mice [35]. Spore treatment alone caused a moderate but significant tumour growth delay, potentially due to modest tumour lysis and haemorrhagic necrosis. However, spore treatment combined with administration of high-dose CB1954 significantly increased the anti-tumour effect; these signs of in vivo efficacy established proof of concept for use of the nitroreductase-CDEPT approach. Theys and colleagues also showed that clostridia can be eliminated with the use of the antibiotic metronidazole, a nitro-prodrug that lacks a bystander effect. This is an important safety aspect as it allows control over the presence of bacteria and therefore control over therapeutic gene expression as the gene is being expressed in the bacterial host. This finding also emphasises the need to employ an enzyme prodrug pairing that generates a substantial bystander effect, to avoid sterilising the clostridial vector during CDEPT.

As the expression level of the therapeutic gene is critical in achieving anti-tumour efficacy with cancer gene therapy, a later study looked at optimising the heterologous gene expression system used in *C. sporogenes* for CDEPT [36]. Two stronger, constitutively expressed, endogenous clostridial promoters (derived from the *abrBP* and *thlP* genes) were found to maximise gene expression levels and replaced the ferredoxin promoter construct used in previous studies. Artificially synthesising the *E. coli nfsB* gene using preferred clostridial codon usage further increased gene expression. By combining the above changes, expression of the nitroreductase was increased by approximately 20-fold compared with the original construct [34,36]. With this new expression system it was shown that previously ineffective doses of CB1954 and PR-104 in vivo could be converted into successful anti-tumour therapy following colonisation of tumours with nitroreductase-expressing *C. sporogenes* [34,36].

In another approach to enhance therapeutic gene expression, combination treatment with vascular disrupting agents to enhance tumour colonisation by clostridia was investigated. These agents specifically target the dividing endothelial cells of tumour blood vessels and cause rapid shut down of blood flow in tumours, promoting hypoxia, anoxia, and necrosis [37]. Animals treated with the combination of Combretastatin A-4 phosphate (CombreAp) and CD-expressing *C. acetobutylicum* spores showed increased levels of vegetative cells and CD expression, presumably as a result of enlarged tumour necrosis and subsequently improved tumour colonisation [32]. Improved tumour colonisation was also seen using Dolastatin-10 [24] and 5,6-dimethylxantheone-4-acetic acid (vadimezan) [12]. These results clearly demonstrate that combined treatment might increase the therapeutic dose intensity and warrants further investigation.

Critically, the construction of a *C. sporogenes* strain that is appropriate for clinical administration was recently reported [38]. Here, Minton and colleagues demonstrate that DNA integration at the *pyrE* locus of *Clostirdium* can be achieved in a series of steps, opening the way for reliable integration of heterologous genes into the clostridial genome. Single-crossover clones harbouring the integrated plasmid are selected with thiamphenicol containing medium and subsequent plasmid excision (a second recombination event to yield double-crossover clones) are selected using 5-fluoroorotic acid. Thus, using carefully designed regional homology, selection of stable double-crossover clones containing large DNA sequences is possible.

Using this technological advance, the DNA open reading frame encoding *E. coli* NfsB was stably integrated into the host chromosome (rather than carried by an ectopic plasmid) [39]. The final expression cassette used did not contain an antibiotic resistance marker, and the strain was disabled for safety and containment by deletion of the *pyrE* gene (the site of transgene insertion) to make it a uracil auxotroph. The authors also identified a novel NfsB nitroreductase from *Neisseria meningitidis* (NmeNTR) that could activate CB1954. The combination of CB1954 and spores expressing NmeNTR or *E. coli* NfsB demonstrated substantial tumour regression in vivo in a subcutaneous HCT116 xenograft model, with some animals being cured.

### 4.2. Advantages of Clostridial Vectors over Viral Delivery Systems

Oncolytic viruses are tumour-specific viruses that infect, replicate in, and lyse tumour cells, resulting in the spread of progeny virus particles to adjacent tumour cells whereby the oncolytic process is repeated [40]. They can directly kill tumour cells as a consequence of the lytic cycle, but like clostridia, can also be ‘armed’ with therapeutic genes incorporated into the viral genome for efficient delivery to tumour tissue. Oncolytic viruses as gene delivery vectors are not without their challenges, however. They typically have the disadvantage of being immunogenic; the neutralisation by antibodies and complement remains a substantial impediment to systemic administration and clinical efficacy [41,42]. In addition, it is often not possible to perform adequate toxicological testing in preclinical animal species because of differences in host tropism [43]. The lytic cycle of the virus may also result in loss of the therapeutic enzyme and/or intracellular co-factors to the extracellular space, and consequently there will be a rim of virus-infected cells that are expressing the gene of interest at any given time which is not ideal from a GDEPT perspective. Clostridia have several desirable features as gene therapy vectors which differentiate them from the oncolytic viruses. These include motility (to facilitate intratumoural spread), a large genome size (to enable insertion of one or more therapeutic genes without restriction on gene length), and the ability to control proliferation with antibiotics. In addition, the bacterial vector genome is autonomous, thus there is minimal risk of recombination with the human host’s genome [44]. Bacteria also multiply faster than mammalian cells, quickly depleting nutrients within the tumour microenvironment. This can affect not only tumour cells but also the tumour-associated cells in the tissue stroma [45].

## 5. The Requirement for Non-invasive Imaging to Expedite Clinical Development

### 5.1. Imaging of Necrosis and Colonisation with *C. sporogenes* in Preclinical Animal Models

The prevalence and quantity of tumour necrosis is essential to the ability of clostridial spores to colonise tumours. In the present preclinical literature there is minimal quantification as to (i) the extent of tumour necrosis required for successful tumour colonisation of clostridial spores and (ii) the amount and distribution of spore colonisation in the tumour necrosis that is required to achieve a therapeutic benefit. Typically, the presence of germinated bacteria is confirmed by Gram-Twort staining, but mostly only high magnification sections of small regions of colonised tumour tissue are displayed [33,39]. Other methods used to confirm the presence of germinated bacteria in tumour tissue include immunoblotting for the inserted gene of interest in excised and homogenised tumours [34,36], and/or using tumour homogenates to carry out an in vitro clostridial growth assay to determine the number of colony forming units (CFU) per gram of tumour tissue [35,39]. More direct imaging was achieved by Liu and colleagues using the nitro-quenched fluorescent probe 6-chloro-9-nitro-5-oxo-5*H*benzo (*a*) phenoxazine (CNOB) to detect the expression of *E. coli nfsB*-labelled *C. sporogenes* spores in subcutaneous murine SiHa xenografts [34]. As the activated probe emits light at the far-red end of the visible spectrum (625 nm) it is capable of low-level tissue penetration, enabling imaging of *C. sporogenes* colonisation and *nfsB* expression levels in vivo. Overall, despite these efforts it appears that the relationship between tumour necrosis and colonisation of this necrosis by clostridial spores is not well defined.

Development and improvement of methods to image the extent of *C. sporogenes* colonisation of tumours would be highly desirable in a preclinical setting. Ideally, development of a non-invasive imaging modality suitable for both preclinical and clinical utility would be preferred. Such a technique would help to quickly and reliably confirm the suitability of the in vivo xenograft model to evaluate efficacy. It would also enable correlation of the strength of signal with the known bacterial titre and/or prodrug efficacy. Information obtained from experiments such as these would thus provide crucial insight into the extent of tumour necrosis required for successful tumour colonisation of clostridial spores, and the amount and distribution of spore colonisation in the tumour necrosis that is required to achieve a therapeutic benefit.

### 5.2. Non-Invasive Imaging of CDEPT Using Positron Emission Tomography

For safety reasons there is a clear need to develop technologies to monitor the spatial and temporal distribution of vector systems with time in a manner that is predictive of normal tissue toxicity and anti-tumour efficacy. Clinically-ready approaches have obvious commercial advantage. For a dynamic agent that amplifies and redistributes over time repeat sample analysis may be necessary, and mandates a non-invasive methodology that can be applied at regular intervals. This is critical to allow early intravenous administration of novel vectors in human clinical trials.

Clinically, magnetic resonance (MR) imaging has evolved as a main diagnostic method used for the pre-operative staging of tumours. However, conventional unenhanced MR imaging is limited in providing detailed information about the extent of tumour necrosis and the presence and/or amount of viable tissue. More recently, several enhanced MR techniques have been used to detect tumour necrosis, including dynamic contrast-enhanced imaging, diffusion weighted imaging, *T_2_*-weighted spin echo imaging, and contrast-enhanced imaging with contrast agents such as Gadophrin-3, which shows great affinity for necrotic tissue [46].

Early clinical endpoints employing pharmacodynamic biomarkers, particularly non-invasive methods, are becoming standard as supporting evidence in the transition from Phase I/II to Phase III clinical trials. Bacteriolytic therapy will face similar hurdles whereby it will be necessary to accurately determine vector distribution and amplification in a spatiotemporal, routine manner for progression through clinical trials. At present, slow incremental modifications to study designs are required for vector development, restricting the pace of clinical progress. Examples include the staged development of the adenoviral vector ONYX-015 and the vaccinia vector JX-594, which required considerable evaluation by localised injection before systemic administration was permitted [47]. Thus, functional imaging of replicating vectors represents an important tool to accelerate clinical development (particularly in the context of systemic administration), allowing monitoring of the location(s), magnitude, and time-variation of replication, in addition to possible early measurements of efficacy and safety. With the introduction of tomographic scanners, such as that used in positron emission tomography (PET) [48], the ability to non-invasively image replicating vectors is now possible [49].

Although no work has yet been published describing PET reporter systems specifically for imaging *Clostridium*, there are a number of systems that have been developed to image viruses that may also have application for imaging bacterial GDEPT vectors. To date, the only clinically approved PET tracer suitable for reporter gene-based imaging is the Sodium Iodide Symporter (hNIS). hNIS is an intrinsic membrane protein that takes up iodide from the extracellular fluid into the cytosol in exchange for sodium. Transfer of the hNIS to gene therapy vectors such as clostridia would allow visualisation with positron emitting radioisotopes such as ^123^I (single-photon emission computed tomography; SPECT) and ^124^I (PET) which are accumulated by hNIS [50]. A high level of transport activity consistently provides a window of sensitivity for the detection of hNIS-mediated accumulation of radioiodide [51], and non-invasive imaging of hNIS upon viral gene transfer has been demonstrated as both feasible and safe in experimental animals and humans [52,53]. Another example of a PET reporter gene for non-invasive imaging of in vivo gene transfer and expression is herpes simplex virus type 1 thymidine kinase (HSV1-tk). HSV1-tk can selectively phosphorylate fluorinated ganciclovir (GCV) analogues which remain entrapped within cells that are subsequently imaged by PET [54]. 9-(4-[^18^F]-fluoro-3-[hydroxymethyl]butyl)-guanine ([^18^F]-FHBG) is an example of a metabolite that is trapped inside transduced cells and detectable as early as two hours after administration [55]. Preclinical safety evaluation has led to the approval of [^18^F]-FHBG as the first investigational new imaging reporter probe by the U.S. Food and Drug Administration (FDA) [56], and studies in humans have demonstrated accumulation of [^18^F]-FHBG in tumour nodules treated by direct injection of an adenoviral vector encoding HSV-tk [57]. However, efficient oncolytic viruses might be difficult to image as tumour cells have limited time to produce hNIS or to trap [^18^F]-FHBG and concentrate the radiotracer before the cell is lysed by the virus. In principle, this would not be a problem for clostridia, which germinate autonomously in the necrotic areas of tumours.

**Figure 3:**
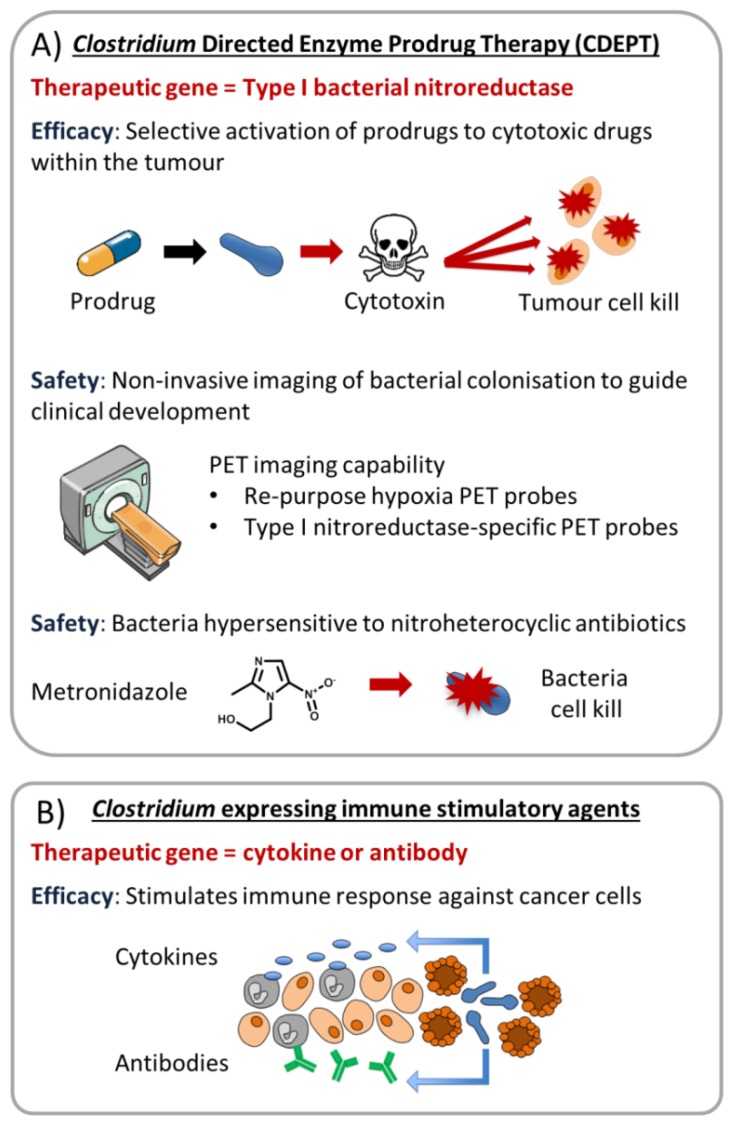
Expression of therapeutic transgenes confers new properties to the bacteria. (**A**) Use of a Type I bacterial nitroreductase has the potential to provide multi-functional features including conditional cytotoxicity, positron emission tomography (PET) imaging capability, and antibiotic hypersensitivity, as discussed previously by Williams and colleagues [60]; (**B**) Use of antibody or cytokine expression to target the tumour microenvironment.

PET probes able to penetrate into human tumours and detect a distant subset of hypoxic cells clearly have adequate pharmacokinetic and pharmacodynamic properties for molecular imaging, and, as such, the probes have attained a high level of validation [48,58,59]. Identification of an optimal nitroreductase ‘reporter gene’ for application in combination with a clinical stage hypoxia PET probe is attractive given all clinical-enabling processes related to the PET probe molecule are a posteriori achieved (Figure 3A). The principle caveat is whether a nitroreductase-expressing vector can be quantified accurately against a background of non-specific probe retention [60].

## 6. Clostridia and Immunotherapy

Activation of the immune system as a treatment modality for cancer has long been a goal in oncology. Although insight into anti-tumour immune responses has significantly increased in recent times, development of immunotherapy for therapeutic benefit in cancer treatment has faced several difficulties, including defining the optimal dose/schedule/patient population and inadequate methods for evaluating efficacy. However, recent proof of clinical efficacy studies in patients treated with several immunotherapy drugs has increased clinical development of cancer immunotherapy [61,62].

Clostridial endospores are themselves poorly immunogenic and fail to elicit an immune response, persisting in multiple organs following intravenous injection [63]. In contrast, once germinated in the necrotic regions of the tumour, vegetative *C. novyi-NT* is immunogenic in syngeneic mouse CT26 colorectal and rabbit VX2 hepatoma tumour models, eliciting a strong inflammatory response with influx of neutrophils and other inflammatory cells [25]. Localised tumour cytokine production leads to monocyte and lymphocyte infiltration accompanied by extensive destruction of tumour tissues. Tumour rechallenge and adoptive transfer experiments demonstrated a CD8+ T cell dependent immunity in *C. novyi-NT* cured subjects, suggesting the innate immune response eventually evolves into an adaptive immune response. These observations suggest a germination-dependent stimulation of an immune response with pathogen-associated antigen recognition acting as an adjuvant.

Clostridia also have the potential to deliver immunotherapy agents directly to tumour tissue [64]. For example, cytokines such as interleukin-2 (IL-2) have been demonstrated to stimulate the immune system [65] and favourable responses to IL-2 therapy have been demonstrated in patients with metastatic melanoma and renal cell cancer [66]. However, high systemic doses of IL-2 are required and administration is associated with a range of dose dependent toxicities [67]. Tumour-selective delivery of IL-2 could potentially negate this problem [68]. *C. acetobutylicum* has previously been engineered to express increased levels of IL-2 and sufficient levels of IL-2 were generated to produce an anti-tumour effect whilst avoiding the side effects demonstrated using systemic administration [69,70]. In addition, small, single chain VHH antibodies generated against HIF-1α have been successfully expressed by an engineered *C. novyi-NT* variant in tumour tissue [71]. Thus, there is great potential to extend these experimental approaches to other cytokines or antibodies applicable to cancer immunotherapy (Figure 3B).

CDEPT may also be combined with cancer immunotherapy. Here, local redistribution of cytotoxic prodrug metabolites results in cell kill in the perinecrotic hypoxic regions, generating local tumour antigen release. Moreover, the hypoxic microenvironment plays an important role in compromising immune cell function, driving immune suppression, and T cell exclusion [72]. Targeting hypoxia in this manner could have a significant effect in altering the properties of the tumour microenvironment, establishing a more immune permissive state.

## 7. Concluding Remarks

Tumour necrosis is most commonly seen in advanced and end-stage cancers where few, if any, treatment options remain. Thus, armed clostridial vectors may represent a valuable salvage therapy when other treatment options are exhausted. Historical clinical evaluation supports proof of concept and recent progress with *C. novyi-NT* confirms the propensity for human tumour colonisation. We propose that non-invasive imaging is fundamental to the successful clinical development of live biotherapeutic agents, as it provides vital real-time biomarker tracking in early safety trials and rational expansion of subsequent efficacy studies. Development of a clostridial vector with PET-imaging capacity could provide a platform technology for the delivery of many biological agents, thereby turning a pathological feature associated with treatment failure into a precision therapy.

## References

[1] Umer B., Good D., Anne J., Duan W., Wei M.Q. (2012). Clostridial spores for cancer therapy: Targeting solid tumour microenvironment. J. Toxicol..

[2] Brown J.M., Giaccia A.J. (1998). The unique physiology of solid tumors: Opportunities (and problems) for cancer therapy. Cancer Res..

[3] Richards C.H., Mohammed Z., Qayyum T., Horgan P.G., McMillan D.C. (2011). The prognostic value of histological tumor necrosis in solid organ malignant disease: A systematic review. Future Oncol..

[4] Martens K., Meyners T., Rades D., Tronnier V., Bonsanto M.M., Petersen D., Dunst J., Dellas K. (2013). The prognostic value of tumor necrosis in patients undergoing stereotactic radiosurgery of brain metastases. Radiat. Oncol..

[5] Fisher E.R., Paleka A., Rockette H., Redmond C., Fisher B. (1978). Pathologic findings from the national surgical ddjuvant breast project (protocol No. 4) V. significance of axillary nodal micro- and macrometastases. Cancer.

[6] Pollheimer M.J., Kornprat P., Lindtner R.A., Harbaum L., Schlemmer A., Rehak P., Langner C. (2010). Tumor necrosis is a new promising prognostic factor in colorectal cancer. Hum. Pathol..

[7] Ladstein R.G., Bachmann I.M., Straume O., Akslen L.A. (2012). Tumor necrosis is a prognostic factor in thick cutaneous melanoma. Am. J. Surg. Pathol..

[8] Swinson D.E., Jones J.L., Richardson D., Cox G., Edwards J.G., O′Byrne K.J. (2002). Tumour necrosis is an independent prognostic marker in non-small cell lung cancer: Correlation with biological variables. Lung Cancer.

[9] Hiraoka N., Ino Y., Sekine S., Tsuda H., Shimada K., Kosuge T., Zavada J., Yoshida M., Yamada K., Koyama T. (2010). Tumour necrosis is a postoperative prognostic marker for pancreatic cancer patients with a high interobserver reproducibility in histological evaluation. Br. J. Cancer.

[10] Sengupta S., Lohse C., Leibovich B., Frank I., Thompson R., Webster W., Zincke H., Blute M.L., Cheville J.C., Kwon E.D. (2005). Histologic coagulative tumor necrosis as a prognostic indicator of renal cell carcinoma aggressiveness. Cancer.

[11] Barbe S., van Mellaert L., Anne J. (2006). The use of clostridial spores for cancer treatment. J. Appl. Microbiol..

[12] Minton N.P. (2003). Clostridia in cancer therapy. Nat. Rev. Microbiol..

[13] Ryan R.M., Green J., Lewis C.E. (2006). Use of bacteria in anti-cancer therapies. Bioessays.

[14] Torrey J.C., Kahn M.C. (1927). The treatment of Flexner-Jobling rat carcinomas with bacterial proteolytic ferments. J. Can. Res..

[15] Connell H.C. (1935). The study and treatment of cancer by proteolytic enzymes: A preliminary report. Can. Med. Assoc. J..

[16] Parker R.C., Plummer H.C., Siebenmann C.O., Chapman M.G. (1947). Effect of Histolyticus Infection and Toxin on Transplantable Mouse Tumors. Proc. Soc. Exp. Biol. Med..

[17] Malmgren R.A., Flanigan C.C. (1955). Localization of the vegetative form of *Clostridium tetani* in mouse tumors following intravenous spore administration. Cancer Res..

[18] Mose J.R., Mose G. (1964). Oncolysis by clostridia. I. Activity of *Clostridium butyricum* (M-55) and other non pathogenic clostridia against the Erlich carcinoma. Cancer Res..

[19] Thiele E.H., Arison R.N., Boxer G.E. (1964). Oncolysis by clostridia. III. Effects of clostridia and chemotherapeutic agents on rodent tumours. Cancer Res..

[20] Engelbart K., Gericke D. (1964). Oncolysis by clostridia. V. Transplanted tumors of the hamster. Cancer Res..

[21] Mohr U., Hondius B.W., Emminger A., Behagel H.A. (1972). Oncolysis by a New Strain of *Clostridium*. Cancer Res..

[22] Carey R.W., Holland J.F., Whang H.Y., Neter E., Bryant B. (1967). Clostridial oncolysis in man. Eur. J. Cancer.

[23] Heppner F., Mose J.R. (1978). The liquefaction (oncolysis) of malignant gliomas by a non pathogenic *Clostridium*. Acta Neurochir..

[24] Dang L.H., Bettegowda C., Huso D.L., Kinzler K.W., Vogelstein B. (2001). Combination bacteriolytic therapy for the treatment of experimental tumors. Proc. Natl. Acad. Sci. USA.

[25] Agrawal N., Bettegowda C., Cheong I., Geshwind J., Drake C.G., Hipkiss E.L., Tatsumi M., Dang L.H., Diaz L.A., Pomper M. (2004). Bacteriolytic therapy can generate a potent immune response against experimental tumors. Proc. Natl. Acad. Sci. USA.

[26] Krick E.L., Sorenmo K.U., Rankin S.U., Cheong I., Kobrin B., Thornton K., Kinzler K.W., Vogelstein B., Zhou S., Diaz L.A. (2012). Evaluation of *Clostridium novyi*-NT spores in dogs with naturally occurring tumors. Am. J. Vet. Res..

[27] Roberts N.J., Zhang L., Janku F., Collins A., Bai R.Y., Staedtke V., Rusk A.W., Tung D., Miller M., Roix J. (2014). Intratumoral injection of *Clostridium novyi*-NT spores induces antitumor responses. Sci. Transl. Med..

[28] McNeish I.A., Searle P.F., Young L.S., Kerr D.J. (1997). Gene directed enzyme prodrug therapy for cancer. Adv. Drug. Del. Rev..

[29] Freytag S.O., Stricker H., Pegg J., Paielli D., Pradhan D.G., Peabody J., de Peralta-Venturina M., Xia X., Brown S., Lu M. (2003). Phase I study of replication-competent adenovirus-mediated double-suicide gene therapy in combination with conventional-dose three-dimensional conformal radiation therapy for the treatment of newly diagnosed, intermediate- to high-risk prostate cancer. Cancer Res..

[30] Patel P., Young J.G., Mautner V., Ashdown D., Bonney S., Pineda R.G., Collins S.I., Searle P.F., Hull D., Peers E. (2009). A phase I/II clinical trial in localized prostate cancer of an adenovirus expressing nitroreductase with CB1954. Mol. Ther..

[31] Fox M.E., Lemmon M.J., Mauchline M.L., Davis T.O., Giaccia A.J., Minton N.P., Brown J.M. (1996). Anaerobic bacteria as a delivery system for cancer gene therapy: In vitro activation of 5-fluorocytosine by genetically engineered clostridia. Gene Ther..

[32] Theys J., Landuyt W., Nuyts S., van Mellaert L., van Oosterom A., Lambin P., Annea J. (2001). Specific targeting of cytosine deaminase to solid tumors by engineered *Clostridium acetobutylicum*. Cancer Gene Ther..

[33] Lemmon M.J., van Zijl P., Fox M.E., Mauchline M.L., Giaccia A.J., Minton N.P., Brown J.M. (1997). Anaerobic bacteria as a gene delivery system that is controlled by the tumor microenvironment. Gene Ther..

[34] Liu S.C., Minton N.P., Giaccia A.J., Brown J.M. (2002). Anticancer efficacy of systemically delivered anaerobic bacteria as gene therapy vectors targeting tumor hypoxia/necrosis. Gene Ther..

[35] Theys J., Pennington O., Dubois L., Anlezark G.M., Vaughan T., Mengesha A., Landuyt W., Anne J., Burke P.J., Durre P. (2006). Repeated cycles of *Clostridium*-directed enzyme prodrug therapy result in sustained antitumour effects in vivo. Br. J. Cancer.

[36] Liu S.C., Ahn G.O., Kioi M., Dorie M.J., Patterson A.V., Brown J.M. (2008). Optimised *Clostridium*-directed enzyme prodrug therapy improves the antitumor activity of the novel DNA crosslinking agent PR-104. Cancer Res..

[37] Brekken R.A., Li C., Kumar S. (2002). Strategies for vascular targeting in tumors. Int. J. Cancer.

[38] Heap J.T., Ehsaan M., Cooksley C.M., Ng Y.K., Cartman S.T., Winzer K., Minton N.P. (2012). Integration of DNA into bacterial chromosomes from plasmids without a counter-selection marker. Nucleic. Acids Res..

[39] Heap J.T., Theys J., Ehsaan M., Kubiak A.M., Dubois L., Paesmans K., van Mellaert L., Knox R., Kuehne S.A., Lambin P. (2014). Spores of *Clostridium* engineered for clinical efficacy and safety cause regression and cure of tumours in vivo. Oncotarget.

[40] Vile R., Ando D., Kirn D. (2002). The oncolytic virotherapy treatment platform for cancer: unique biological and biosafety points to consider. Cancer Gene Ther..

[41] Cattaneo R., Miest T., Shashkova E.V., Barry M.A. (2008). Reprogrammed viruses as cancer therapeutics: targeted, armed and shielded. Nat. Rev. Microbiol..

[42] Parato K.A., Senger D., Forsyth P.A., Bell J.C. (2005). Recent progress in the battle between oncolytic viruses and tumours. Nat. Rev. Cancer.

[43] Patel M.R., Kratzke R.A. (2013). Oncolytic virus therapy for cancer: The first wave of translational clinical trials. Transl. Res..

[44] Hawkins L.K., Lemoine N.R., Kirn D. (2002). Oncolytic biotherapy: a novel therapeutic platform. Lancet Oncol..

[45] Wei M.Q., Mengesha A., Good D., Anne J. (2008). Bacterial targeted tumour therapy—Dawn of a new era?. Cancer Lett..

[46] Egeland T.A.M., Gaustad J.V., Galante Y.M., Rofstad E.K. (2011). Magnetic resonance imaging of tumour necrosis. Acta Oncol..

[47] Kirn D., Martuza R.L., Zwiebel J. (2001). Replication-selective virotherapy for cancer: Biological principles, risk management and future directions. Nat. Med..

[48] Gambhir S.S. (2002). Molecular imaging of cancer with positron emission tomography. Nat. Rev. Cancer.

[49] Min J.J., Gambhir S.S. (2004). Gene therapy progress and prospects: noninvasive imaging of gene therapy in living subjects. Gene Ther..

[50] Ahn B. (2012). Sodium Iodide Symporter for Nuclear Molecular Imaging and Gene Therapy: From Bedside to Bench and Back. Theranostics.

[51] Penheiter A.R., Russell S.J., Carlson S.K. (2012). The Sodium Iodide Symporter (NIS) as an Imaging Reporter for Gene, Viral, and Cell-based Therapies. Curr. Gene Ther..

[52] Barton K.N., Stricker H., Brown S.L., Elshaikh M., Aref I., Lu M., Pegg J., Zhang Y., Karvelis K.C., Siddiqui F. (2008). Phase I study of noninvasive imaging of adenovirus-mediated gene expression in the human prostate. Mol. Ther..

[53] Msaouel P., Dispenzieri A., Galanis E. (2009). Clinical testing of engineered oncolytic measles virus strains in the treatment of cancer: An overview. Curr. Opin. Mol. Ther..

[54] Bhaumik S. (2011). Advances in Imaging Gene-Directed Enzyme Prodrug Therapy. Curr. Pharm. Biotechnol..

[55] Kuruppu D., Brownell A.L., Zhu A., Yu M., Wang X., Kulu Y., Fuchs B.C., Kawasaki H., Tanabe K.K. (2007). Positron Emission Tomography of Herpes Simplex Virus 1 Oncolysis. Cancer Res..

[56] Yaghoubi S.S., Couto M.A., Chen C.C., Polavaram L., Cui G., Sen L., Gambhir S.S. (2006). Preclinical safety evaluation of 18F-FHBG: A pet reporter probe for imaging herpes simplex virus type 1 thymidine kinase (HSV1-tk) or mutant HSV1-sr39tk’s expression. J. Nucl. Med..

[57] Penuelas I., Mazzolini G., Boan J.F., Sangro B., Marti-Climent J., Ruiz M., Ruiz J., Satyamurthy N., Qian C., Barrio J.R. (2005). Positron emission tomography imaging of adenoviral-mediated transgene expression in liver cancer patients. Gastroenterology.

[58] Peeters S.G., Zegers C.M., Lieuwes N.G., van Elmpt W., Eriksson J., van Dongen G.A., Dubois L., Lambin P. (2015). A comparative study of the hypoxia PET tracers [(1)(8)F]HX4, [(1)(8)F]FAZA, and [(1)(8)F]FMISO in a preclinical tumor model. Int. J. Radiat. Oncol. Biol. Phys..

[59] Min J.J., Gambhir S.S. (2008). Molecular imaging of PET reporter gene expression. Handb. Exp. Pharmacol..

[60] Williams E.M., Little R.F., Mowday A.M., Rich M.H., Chan-Hyams J.V., Copp J.N., Smaill J.B., Patterson A.V., Ackerley D.F. (2015). Nitroreductase gene-directed enzyme prodrug therapy: Insights and advances toward clinical utility. Biochem. J..

[61] Hodi F.S., O′Day S.J., McDermott D.F., Weber R.W., Sosman J.A., Haanen J.B., Gonzalez R., Robert C., Schadendorf D., Hassel J.C. (2010). Improved survival with ipilimumab in patients with metastatic melanoma. N. Eng. J. Med..

[62] Brahmer J.R., Tykodi S.S., Chow L.Q., Hwu W.J., Topalian S.L., Hwu P., Drake C.G., Camacho L.H., Kauh J., Odunsi K. (2012). Safety and Activity of Anti–PD-L1 Antibody in Patients with Advanced Cancer. N. Eng. J. Med..

[63] Fabricius E.M., Schneeweiss U., Schau H.P., Schmidt W., Benedix A. (1993). Quantitative investigations into the elimination of in vitro-obtained spores of the non-pathogenic *Clostridium butyricum* strain CNRZ 528, and their persistence in organs of different species following intravenous spore administration. Res. Microbiol..

[64] Nuyts S., van Mellaert L., Theys J., Landuyt W., Bosmans E., Anne J., Lambin P. (2001). Radio-responsive recA promoter significantly increases TNFalpha production in recombinant clostridia after 2 Gy irradiation. Gene Ther..

[65] Antony G.K., Dudek A.Z. (2010). Interleukin 2 in cancer therapy. Curr. Med. Chem..

[66] Rosenberg S.A., Yang J.C., Topalian S.L., Schwartzentruber D.J., Weber J.S., Parkinson D.R., Seipp C.A., Einhorn J.H., White D.E. (1994). Treatment of 283 consecutive patients with metastatic melanoma or renal cell cancer using high dose bolus IL-2. JAMA.

[67] Lotze M.T., Matory Y.L., Rayner A.A., Ettinghausen S.E., Vetto J.T., Seipp C.A., Rosenberg S.A. (1986). Clinical effects and toxicities of IL-2 in patients with cancer. Cancer.

[68] Zegers C.M., Rekers N.H., Quaden D.H., Lieuwes N.G., Yaromina A., Germeraad W.T., Wieten L., Biessen E.A., Boon L., Neri D. (2015). Radiotherapy combined with the immunocytokine L19-IL2 provides long-lasting antitumor effects. Clin. Cancer Res..

[69] Theys J., Landuyt W., Nuyts S., van Mellaert L., Bosmans E., Rijnders A., van Den Bogaert W., van Oosterom A., Anne J., Lambin P. (2001). Improvement of clostridium tumour targeting vectors evaluated in rat rhabdomyosarcomas. FEMS Immunol. Med. Microbiol..

[70] Barbe S., van Mellaert L., Theys J., Geukens N., Lammertyn E., Lambin P., Anne J. (2005). Secretory production of biologically active rat interleukin-2 by *Clostridium acetobutylicum* DSM792 as a tool for anti-tumor treatment. FEMS Microbiol. Lett..

[71] Groot A.J., Mengesha A., van der Wall E., van Diest P.J., Theys J., Vooijs M. (2007). Functional antibodies produced by oncolytic clostridia. Biochem. Biophys. Res. Commun..

[72] Hasan A., Mazzone M. (2015). Sixty shades of oxygen—An attractive opportunity for cancer immunotherapy. Ann. Transl. Med..

